# Trends in Methadone Dispensing for Opioid Use Disorder After Medicare Payment Policy Changes

**DOI:** 10.1001/jamanetworkopen.2023.14328

**Published:** 2023-05-19

**Authors:** Erin A. Taylor, Jonathan H. Cantor, Ashley C. Bradford, Kosali Simon, Bradley D. Stein

**Affiliations:** 1 RAND Corporation, Santa Monica, California; 2 The Paul H. O’Neill School of Public and Environmental Affairs, Indiana University, Bloomington; 3 RAND Corporation, Pittsburgh, Pennsylvania

## Abstract

**Importance:**

A significant proportion of Medicare beneficiaries have a diagnosed opioid use disorder (OUD). Methadone and buprenorphine are both effective medications for the treatment of OUD (MOUDs); however, Medicare did not cover methadone until 2020.

**Objective:**

To examine trends in methadone and buprenorphine dispensing among Medicare Advantage (MA) enrollees after 2 policy changes in 2020 related to methadone access.

**Design, Setting, and Participants:**

This cross-sectional analysis of temporal trends in methadone and buprenorphine treatment dispensing assessed MA beneficiary claims from January 1, 2019, through March 31, 2022, captured by Optum’s Clinformatics Data Mart. Of 9 870 791 MA enrollees included in the database, 39 252 had at least 1 claim for methadone, buprenorphine, or both during the study period. All available MA enrollees were included. Subanalyses by age and dual eligibility for Medicare and Medicaid status were conducted.

**Exposures:**

Study exposures were (1) the Centers for Medicare & Medicaid Services (CMS) Medicare bundled payment reimbursement policy for OUD treatment and (2) the Substance Abuse and Mental Health Administration and CMS Medicare policies designed to facilitate access to treatment for OUD, specifically during the COVID-19 pandemic.

**Main Outcomes and Measures:**

Study outcomes were trends in methadone and buprenorphine dispensing by beneficiary characteristics. National methadone and buprenorphine dispensing rates were calculated as claims-based dispensing rates per 1000 MA enrollees.

**Results:**

Among the 39 252 MA enrollees with at least 1 MOUD dispensing claim (mean age, 58.6 [95% CI, 58.57-58.62] years; 45.9% female), 195 196 methadone claims and 540 564 buprenorphine pharmacy claims were identified, for a total of 735 760 dispensing claims. The methadone dispensing rate for MA enrollees was 0 in 2019 because the policy did not allow any payment until 2020. Claims rates per 1000 MA enrollees were low initially, increasing from 0.98 in the first quarter of 2020 to 4.71 in the first quarter of 2022. Increases were primarily associated with dually eligible beneficiaries and beneficiaries younger than 65 years. National buprenorphine dispensing rates were 4.64 per 1000 enrollees in quarter 1 of 2019, increasing to 7.45 per 1000 enrollees in quarter 1 of 2022.

**Conclusions and Relevance:**

This cross-sectional study found that methadone dispensing increased among Medicare beneficiaries after the policy changes. Rates of buprenorphine dispensing did not provide evidence that beneficiaries substituted buprenorphine for methadone. The 2 new CMS policies represent an important first step in increasing access to MOUD treatment for Medicare beneficiaries.

## Key Points

**Question:**

Did rates of methadone dispensing for opioid use disorder increase after Medicare payment and COVID-19 policy changes in 2020, and did these rates vary by beneficiary age and dual eligibility for Medicaid?

**Findings:**

In this cross-sectional study of 9 870 791 Medicare Advantage enrollees, increased rates of methadone dispensing were observed, largely driven by beneficiaries dually eligible for Medicare and Medicaid and those younger than 65 years.

**Meaning:**

These findings suggest that policies designed to increase access to methadone treatment appear to have increased access to medication treatment for Medicare beneficiaries with opioid use disorder, helping to meet national policy priorities.

## Teaser

This cross-sectional study explores changes in methadone and buprenorphine dispensing after the implementation of Medicare policy changes regarding payment and access in 2020.

## Introduction

The ongoing opioid crisis in the US resulted in an estimated 80 411 overdose deaths in 2021 alone.^1^ Medications for opioid use disorder (MOUDs) are an important treatment for individuals with an opioid use disorder (OUD).^2,3,4,5,6^ These medications include buprenorphine, most commonly available in tablet or film form, prescribed in an office-based setting, and consumed like other medications for chronic conditions; naltrexone, which is available as a take-home prescription or injection but requires some extended period without opioids before initiation; and methadone, a schedule II opioid that can be taken at any point in OUD treatment but is dispensed only to patients registered with a federally certified and licensed opioid treatment program (OTP).^7^ All these medications have been shown to be safe and effective in treating OUD, and methadone in particular is one of the most effective treatments.^8^ However, until January 2020, the Medicare Program, one of the largest health care payers in the US, did not cover methadone as an option for treatment of OUD.^9,10^ Less than 16% of the 1 million Medicare beneficiaries with OUD received any kind of MOUD in 2020.^11,12^ One reason for the low rate of MOUD use by Medicare beneficiaries is that Medicare did not cover methadone for the treatment of OUD before 2020.

In January 2020, the Centers for Medicare & Medicaid Services (CMS) implemented a new Medicare bundled payment reimbursement policy for OUD treatment^13^ that, for the first time, included methadone treatment. Opioid treatment programs must be certified by the Substance Abuse and Mental Health Services Administration (SAMHSA) and registered with the CMS to receive payment for providing OUD treatment for Medicare beneficiaries^14^ and be eligible to receive the payments. Medicare beneficiaries receiving coverage through traditional Medicare and those who chose to enroll in Medicare Advantage (MA) plans—which provide Medicare coverage but are administered by private insurance companies—were newly able to access methadone as a treatment option as part of this new policy. The new bundled payment policy was supplemented in March 2020 with SAMHSA and CMS Medicare policy changes designed to facilitate access to treatment for OUD, including access to take-home methadone through OTPs, during the COVID-19 pandemic.^15^

The expanded Medicare coverage of OUD treatment to include methadone could have important effects on access to care, both in terms of access to the newly covered methadone and for other types of MOUDs. We explored the effect of these policy changes by examining the trends in methadone dispensing beginning in 2020, when Medicare began coverage of methadone for treatment of OUD. We were able to track the trend of buprenorphine dispensing with a longer look-back period, beginning in 2019. We tracked quarterly trends in MA dispensing for both types of MOUDs through March 2022. We also examined trends separately for beneficiaries of different ages as well as for beneficiaries dually eligible for Medicare and Medicaid, a group with high rates of OUD.^16^

## Methods

This cross-sectional study used Optum’s Clinformatics Data Mart (CDM), which is derived from a database of administrative health claims for members of large commercial and MA plans and has previously been used to examine methadone dispensing.^17^ Methadone may only be dispensed by certified OTPs and is billed as part of Medicare Part B, so we used the associated Healthcare Common Procedure Coding System (HCPCS) codes^18^ to identify methadone treatment services for OUD in each quarter from January 1, 2020, through March 31, 2022. Because methadone was not covered by Medicare before 2020, we were not able to calculate the rates of dispensing for Medicare beneficiaries before the policy was implemented. As part of the COVID-19 pandemic, take-home methadone was also added as an option, allowing us to calculate the rates of take-home methadone (HCPCS code G2078) dispensed and compare those rates to methadone dispensed and taken at an OTP (HCPCS code G2067). Although we have access to race and ethnicity data as part of the claims data, we report on the claims dispensing rates for beneficiaries at the national level, by age, and by dual eligibility status to focus on our initial study. Future studies will explore differences in treatment by race and ethnicity. Indiana University’s institutional review board deemed the research to be exempt because it does not involve human participants; therefore, no informed consent was required. This study follows the Strengthening the Reporting of Observational Studies in Epidemiology (STROBE) reporting guideline for cross-sectional studies.

### Statistical Analysis

Of 9 870 791 MA enrollees included in the database, 39 252 MA enrollees had at least 1 claim for methadone, buprenorphine, or both during our study period. We included all beneficiaries enrolled in MA plans at any time in 2019 through 2022. Rates were calculated within each quarter as the number of claims per 1000 MA enrollees. To better understand disparities in access to treatment, we stratified the rates by age (≥65 and <65 years) and by dual eligibility for Medicare and Medicaid.

The new coverage of methadone by Medicare may have led to substitution away from other types of MOUDs, especially buprenorphine. To compare the rates of buprenorphine dispensing with the methadone claims rates, we used the CDM data to construct national quarterly rates of buprenorphine dispensing at retail pharmacies for MA enrollees from 2019 through 2022. There were very few buprenorphine dispensing claims at OTPs, which would be billed under Medicare Part B; therefore, we excluded them from analyses. eTable 1 in Supplement 1 gives the buprenorphine codes used for these analyses, and eTables 2 through 4 in Supplement 1 provide the data underlying each figure. All analyses were completed using Stata software, version 17.0 (StataCorp LLC).

## Results

**Figure 1.**
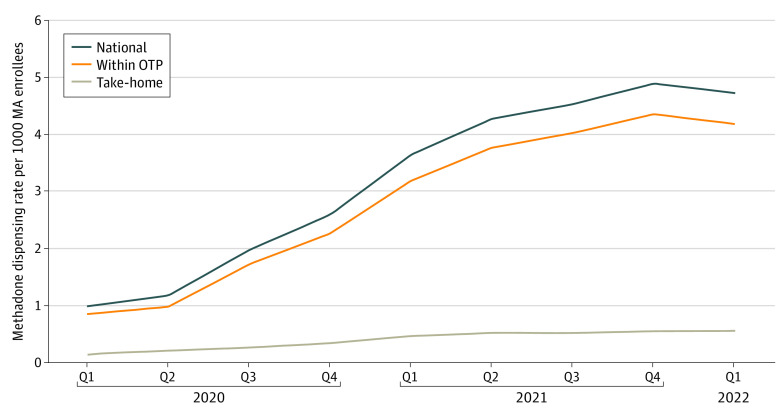
Methadone Dispensing Rates per 1000 Medicare Advantage (MA) Enrollees by Dispensing Type OTP indicates opioid treatment program; Q, quarter.

Among the 39 252 enrollees with at least 1 MOUD claim, we captured 195 196 methadone claims and 540 564 buprenorphine pharmacy claims, for a total of 735 760 dispensing claims. The mean age of MA enrollees with at least 1 MOUD dispensing claim was 58.59 (95% CI, 58.57-58.62) years; 45.90% (95% CI, 45.79%-46.02%) were female and 54.10% (95% CI, 53.98%-54.21%) were male. The methadone dispensing rate for MA enrollees was 0 in 2019 because the policy did not allow any payment until 2020. Claims rates per 1000 MA enrollees were low initially, increasing from 0.98 in the first quarter of 2020 to 4.71 in the first quarter of 2022 (Figure 1). Methadone service use was substantially greater for methadone dispensed and consumed at the OTP, with rates for within-OTP dispensing starting at 0.84 and increasing to 4.17 during the same period. Take-home methadone rates started very low (0.14) and increased at a slower rate compared with the national rate and the within-OTP rate.

**Figure 2.**
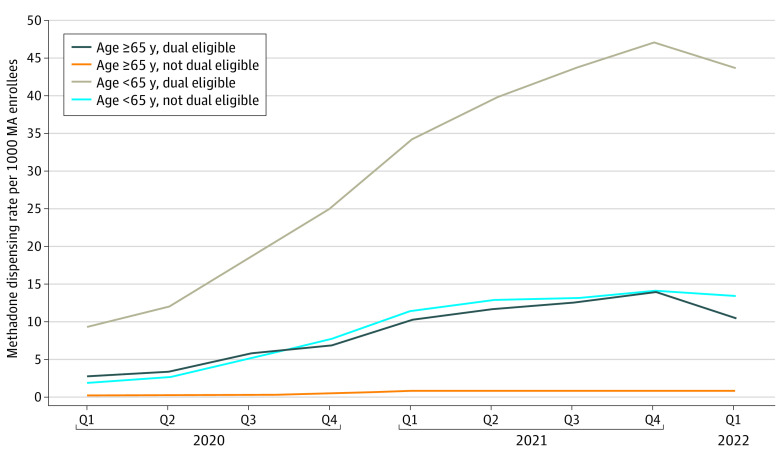
Methadone Dispensing Rates per 1000 Medicare Advantage (MA) Enrollees by Age and Dual Eligibility Status Q indicates quarter.

Of the 4 groups we studied, methadone service use was primarily associated with dually eligible beneficiaries younger than 65 years, whereas beneficiaries younger than 65 years who were not dually eligible had the second-highest rate (Figure 2). Dually eligible beneficiaries 65 years or older had rates substantially higher than the rates for individuals 65 years or older who were not dually eligible.

**Figure 3.**
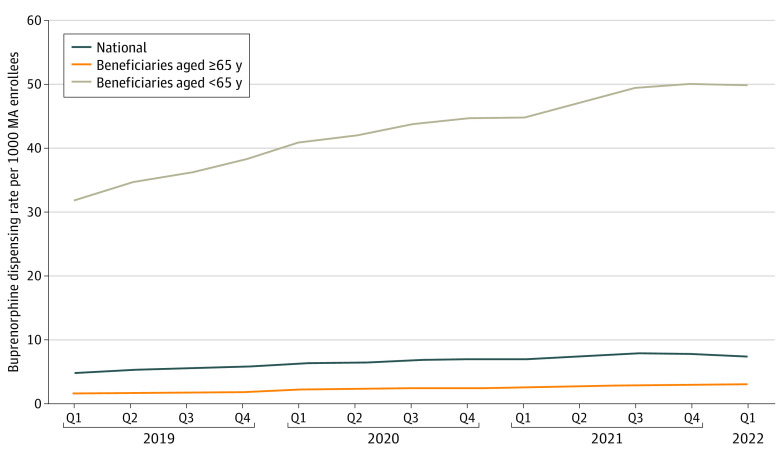
Rates of Buprenorphine Dispensed From Pharmacies per 1000 Medicare Advantage (MA) Enrollees by Age Q indicates quarter.

Rates of buprenorphine dispensed from pharmacies to MA enrollees were fairly low and gradually increased both before the implementation of the new methadone coverage policy and in the 2 years after (Figure 3). National buprenorphine dispensing rates were 4.64 in quarter 1 of 2019, increasing to 7.45 in quarter 1 of 2022. When splitting these rates by age categories, however, the rates for beneficiaries 65 years or older were very low, ranging from 1.53 to 2.92, whereas rates for beneficiaries younger than 65 years started very high (31.68) in quarter 1 of 2019 and increased to 50.0 by quarter 1 of 2022.

## Discussion

The implementation of the Medicare payment policy change as well as the policies designed to increase access to OUD treatment during the COVID-19 pandemic appear to be associated with increasing rates of methadone services in each quarter after the policy went into effect in January 2020. Rates of methadone services accelerated after the second quarter of 2020. Our findings suggest a strong uptake by beneficiaries younger than 65 years, largely driven by dually eligible beneficiaries, who represented approximately 22% to 26% of all MA enrollees younger than 65 years. The rate of increase slowed or even reversed in the most recent quarter for all groups. Future work should explore reasons for the differential increases across MA enrollees by dual eligibility status and by age as well as whether there are similar rates of use by Medicare beneficiaries enrolled in fee-for-service Medicare.

Although methadone plays an important role in the treatment of OUD, methadone treatment receipt is not recorded in many data sources or in other survey data sets. Surveys are, however, conducted at OTP facilities to record methadone administration.^19^ A series of surveys^20,21,22^ conducted via National Drug and Alcohol Testing Systems in 2008 to 2017 documented both dosage patterns in methadone use and found that many practitioners dose underrecommended levels, particularly in racially underrepresented populations. However, the National Survey of Substance Abuse Treatment Services and National Drug and Alcohol Testing Systems do not have data on individual patients. Data on individuals’ receipt of methadone for treatment come only from claims data, to the best of our knowledge.

### Limitations

This study is not without limitations. The primary limitation is that beneficiaries may have received methadone covered by other insurance options (eg, Medicaid) or block grants before the Medicare change, with some observed increases in methadone services for those younger than 65 years and dually eligible beneficiaries, representing a possible shift of payer to Medicare. Although further research would be needed to understand the effects of substituting across payers, this study contributes to existing knowledge by providing early data on uptake of methadone by MA beneficiaries after the payment policy change. A second limitation is that these data are for a subset of enrollees in MA plans offered by one of the largest MA insurers, and therefore results may not be generalizable to the broader Medicare population. However, MA represents an increasingly large share of Medicare enrollment.^23^ Claims data provide no information on the severity of an individual’s opioid use disorder, decisions regarding the use of MOUDs and choice of medication, or clinical outcomes that result from treatment.

## Conclusions 

Despite these limitations, increasing access to effective treatment for OUD is a national priority,^24^ and this initial look at dispensing rates after a new Medicare policy suggests the policy may have facilitated access to medication treatment for Medicare beneficiaries with OUD during the COVID-19 pandemic, particularly for dually eligible beneficiaries younger than 65 years. The relatively steady rate of increase in buprenorphine prescribing, which showed no obvious discontinuity after the CMS change to methadone coverage, did not provide evidence that beneficiaries were substituting methadone for buprenorphine. Future research is needed to investigate whether the changes that we document represent an increase in overall OUD treatment and the effects of the policies on clinical outcomes for Medicare beneficiaries with OUD.

## Notes

Supplement 1. **eTable 1.** Buprenorphine NDC Codes **eTable 2.** Methadone Dispensing Rates per 1000 MA Enrollees, by Dispensing Type **eTable 3.** Methadone Dispensing Rates per 1000 MA Enrollees, by Age and Dual Eligibility Status **eTable 4.** Rates of Buprenorphine Dispensed From Pharmacies per 1000 MA Enrollees, by Age

Supplement 2.
Data Sharing Statement
