# Supplementary material for: Trends in Methadone Dispensing for Opioid Use Disorder After Medicare Payment Policy Changes
Source: JAMA Netw Open. 2023 May 19;6(5):e2314328. doi: 10.1001/jamanetworkopen.2023.14328 (PMC10199341; doi:10.1001/jamanetworkopen.2023.14328)
Supplement: Supplement 1. — eTable 1. Buprenorphine NDC Codes eTable 2. Methadone Dispensing Rates per 1000 MA Enrollees, by Dispensing Type eTable 3. Methadone Dispensing Rates per 1000 MA Enrollees, by Age and Dual Eligibility Status eTable 4. Rates of Buprenorphine Dispensed From Pharmacies per 1000 MA Enrollees, by Age [file jamanetwopen-e2314328-s001.pdf]

Supplementary Online Content

Taylor EA, Cantor JH, Bradford AC, Simon K, Stein BD. Trends in methadone dispensing for opioid use disorder after Medicare payment policy changes. *JAMA Netw Open*. 2023;6(5):e2314328. doi:10.1001/jamanetworkopen.2023.14328

eTable 1. Buprenorphine NDC Codes
eTable 2. Methadone Dispensing Rates per 1000 MA Enrollees, by Dispensing Type
eTable 3. Methadone Dispensing Rates per 1000 MA Enrollees, by Age and Dual Eligibility Status
eTable 4. Rates of Buprenorphine Dispensed From Pharmacies per 1000 MA Enrollees, by Age

This supplementary material has been provided by the authors to give readers additional information about their work.

© 2023 Taylor EA al. *JAMA Network Open.*

eTable 1. Buprenorphine NDC Codes

NDC	Drug Name	Formulation
54017613	BUPRENORPHINE HYDROCHLORIDE	Tablet
54017713	BUPRENORPHINE HYDROCHLORIDE	Tablet
54018813	BUPRENORPHINE-NALOXONE	Tablet
54018913	BUPRENORPHINE-NALOXONE	Tablet
93537856	BUPRENORPHINE HYDROCHLORIDE	Tablet
93537956	BUPRENORPHINE HYDROCHLORIDE	Tablet
93572056	BUPRENORPHINE-NALOXONE	Tablet
93572156	BUPRENORPHINE-NALOXONE	Tablet
228315303	BUPRENORPHINE HYDROCHLORIDE	Tablet
228315403	BUPRENORPHINE-NALOXONE	Tablet
228315473	BUPRENORPHINE-NALOXONE	Tablet
228315503	BUPRENORPHINE-NALOXONE	Tablet
228315567	BUPRENORPHINE-NALOXONE	Tablet
228315573	BUPRENORPHINE-NALOXONE	Tablet
228315603	BUPRENORPHINE HYDROCHLORIDE	Tablet
378092393	BUPRENORPHINE HYDROCHLORIDE	Tablet
378092493	BUPRENORPHINE HYDROCHLORIDE	Tablet
378876593	BUPRENORPHINE-NALOXONE	Film
378876693	BUPRENORPHINE-NALOXONE	Film
378876716	BUPRENORPHINE-NALOXONE	Film
378876793	BUPRENORPHINE-NALOXONE	Film
378876816	BUPRENORPHINE-NALOXONE	Film
378876893	BUPRENORPHINE-NALOXONE	Film
406192303	BUPRENORPHINE-NALOXONE	Tablet
406192309	BUPRENORPHINE-NALOXONE	Tablet
406192403	BUPRENORPHINE-NALOXONE	Tablet
406192409	BUPRENORPHINE-NALOXONE	Tablet
406800503	BUPRENORPHINE-NALOXONE	Tablet
406802003	BUPRENORPHINE-NALOXONE	Tablet
490005100	SUBOXONE	Tablet
490005130	SUBOXONE	Tablet
490005160	SUBOXONE	Tablet
490005190	SUBOXONE	Tablet
781721664	BUPRENORPHINE-NALOXONE	Film
781722764	BUPRENORPHINE-NALOXONE	Film
781723806	BUPRENORPHINE-NALOXONE	Film
781723864	BUPRENORPHINE-NALOXONE	Film

© 2023 Taylor EA al. *JAMA Network Open.*

NDC	Drug Name	Formulation
781724964	BUPRENORPHINE-NALOXONE	Film
12496010001	SUBLOCADE	Extended Release Subcutaneous
12496010002	SUBLOCADE 100MG	Solution, Extended Release
12496010005	SUBLOCADE 100MG	Solution, Extended Release
12496030001	SUBLOCADE	Extended Release Subcutaneous
12496030002	SUBLOCADE	Extended Release Subcutaneous
12496030005	SUBLOCADE	Extended Release Subcutaneous
12496120201	SUBOXONE	Film
12496120203	SUBOXONE	Film
12496120401	SUBOXONE	Film
12496120403	SUBOXONE	Film
12496120801	SUBOXONE	Film
12496120803	SUBOXONE	Film
12496121201	SUBOXONE	Film
12496121203	SUBOXONE	Film
12496127802	SUBUTEX	Tablet
12496128302	SUBOXONE	Tablet
12496130602	SUBOXONE	Tablet
12496131002	SUBUTEX	Tablet
16590066605	SUBOXONE	Tablet
16590066630	SUBOXONE	Tablet
16590066705	SUBOXONE	Tablet
16590066730	SUBOXONE	Tablet
16590066790	SUBOXONE	Tablet
23490927003	SUBOXONE	Tablet
23490927006	SUBOXONE	Tablet
23490927009	SUBOXONE	Tablet
35356000407	SUBOXONE	Tablet
35356000430	SUBOXONE	Tablet
35356055530	BUPRENORPHINE HYDROCHLORIDE	Tablet
35356055630	BUPRENORPHINE HYDROCHLORIDE	Tablet
42291017430	BUPRENORPHINE-NALOXONE	Tablet
42291017530	BUPRENORPHINE-NALOXONE	Tablet
42858050103	BUPRENORPHINE HYDROCHLORIDE	Tablet
42858050203	BUPRENORPHINE HYDROCHLORIDE	Tablet
43063018407	SUBOXONE	Tablet
43063018430	SUBOXONE	Tablet
43063066706	BUPRENORPHINE HYDROCHLORIDE	Tablet
43063075306	BUPRENORPHINE HYDROCHLORIDE	Tablet

© 2023 Taylor EA al. *JAMA Network Open.*

NDC	Drug Name	Formulation
43598057901	BUPRENORPHINE-NALOXONE	Film
43598057930	BUPRENORPHINE-NALOXONE	Film
43598058001	BUPRENORPHINE-NALOXONE	Film
43598058030	BUPRENORPHINE-NALOXONE	Film
43598058101	BUPRENORPHINE-NALOXONE	Film
43598058130	BUPRENORPHINE-NALOXONE	Film
43598058201	BUPRENORPHINE-NALOXONE	Film
43598058230	BUPRENORPHINE-NALOXONE	Film
47781035503	BUPRENORPHINE-NALOXONE	Film
47781035603	BUPRENORPHINE-NALOXONE	Film
47781035703	BUPRENORPHINE-NALOXONE	Film
47781035803	BUPRENORPHINE-NALOXONE	Film
47781071203	BUPRENORPHINE-NALOXONE	Film
47781071211	BUPRENORPHINE-NALOXONE	Film
49999039507	SUBOXONE	Tablet
49999039515	SUBOXONE	Tablet
49999039530	SUBOXONE	Tablet
49999063830	SUBUTEX	Tablet
49999063930	SUBUTEX	Tablet
50090157100	BUPRENORPHINE HYDROCHLORIDE	Tablet
50090292400	BUPRENORPHINE HYDROCHLORIDE	Tablet
50268014411	BUPRENORPHINE-NALOXONE	Tablet
50268014415	BUPRENORPHINE-NALOXONE	Tablet
50268014511	BUPRENORPHINE-NALOXONE	Tablet
50268014515	BUPRENORPHINE-NALOXONE	Tablet
50383028793	BUPRENORPHINE-NALOXONE	Tablet
50383029493	BUPRENORPHINE-NALOXONE	Tablet
50383092493	BUPRENORPHINE HYDROCHLORIDE	Tablet
50383093093	BUPRENORPHINE HYDROCHLORIDE	Tablet
52427069203	BUPRENORPHINE-NALOXONE	Film
52427069211	BUPRENORPHINE-NALOXONE	Film
52427069403	BUPRENORPHINE-NALOXONE	Film
52427069411	BUPRENORPHINE-NALOXONE	Film
52427069803	BUPRENORPHINE-NALOXONE	Film
52427069811	BUPRENORPHINE-NALOXONE	Film
52440010014	PROBUPHINE	Implant
52959030430	SUBOXONE	Tablet
52959074930	SUBOXONE	Tablet
53217013830	BUPRENORPHINE-NALOXONE	Tablet

© 2023 Taylor EA al. *JAMA Network Open.*

NDC	Drug Name	Formulation
53217024630	BUPRENORPHINE HYDROCHLORIDE	Tablet
54123011430	ZUBSOLV	Tablet
54123090730	ZUBSOLV	Tablet
54123091430	ZUBSOLV	Tablet
54123092930	ZUBSOLV	Tablet
54123095730	ZUBSOLV	Tablet
54123098630	ZUBSOLV	Tablet
54569549600	SUBOXONE	Tablet
54569573900	SUBOXONE	Tablet
54569573901	SUBOXONE	Tablet
54569573902	SUBOXONE	Tablet
54569639900	SUBOXONE	Film
54569640800	BUPRENORPHINE-NALOXONE	Tablet
54569657800	BUPRENORPHINE HYDROCHLORIDE	Tablet
54868570700	SUBOXONE	Tablet
54868570701	SUBOXONE	Tablet
54868570702	SUBOXONE	Tablet
54868570703	SUBOXONE	Tablet
54868570704	SUBOXONE	Tablet
54868575000	SUBOXONE	Tablet
55045378403	SUBOXONE	Tablet
55700014730	SUBOXONE	Film
55700018430	BUPRENORPHINE-NALOXONE	Tablet
55700030230	BUPRENORPHINE HYDROCHLORIDE	Tablet
55700030330	BUPRENORPHINE HYDROCHLORIDE	Tablet
55887031204	SUBOXONE	Tablet
55887031215	SUBOXONE	Tablet
58284010014	PROBUPHINE	Implant
59385001201	BUNAVAIL	Film
59385001230	BUNAVAIL	Film
59385001401	BUNAVAIL	Film
59385001430	BUNAVAIL	Film
59385001601	BUNAVAIL	Film
59385001630	BUNAVAIL	Film
60429058630	BUPRENORPHINE-NALOXONE	Tablet
60429058633	BUPRENORPHINE-NALOXONE	Tablet
60429058730	BUPRENORPHINE-NALOXONE	Tablet
60429058733	BUPRENORPHINE-NALOXONE	Tablet
60687048111	BUPRENORPHINE	Tablet

© 2023 Taylor EA al. *JAMA Network Open.*

NDC	Drug Name	Formulation
60687048121	BUPRENORPHINE	Tablet
60687049211	BUPRENORPHINE	Tablet
60687049221	BUPRENORPHINE	Tablet
60846097003	BUPRENORPHINE-NALOXONE	Tablet
60846097103	BUPRENORPHINE-NALOXONE	Tablet
62175045232	BUPRENORPHINE-NALOXONE	Tablet
62175045832	BUPRENORPHINE-NALOXONE	Tablet
62756045964	BUPRENORPHINE HYDROCHLORIDE	Tablet
62756045983	BUPRENORPHINE HYDROCHLORIDE	Tablet
62756046064	BUPRENORPHINE HYDROCHLORIDE	Tablet
62756046083	BUPRENORPHINE HYDROCHLORIDE	Tablet
62756096964	BUPRENORPHINE-NALOXONE	Tablet
62756096983	BUPRENORPHINE-NALOXONE	Tablet
62756097064	BUPRENORPHINE-NALOXONE	Tablet
62756097083	BUPRENORPHINE-NALOXONE	Tablet
63629403401	SUBOXONE	Tablet
63629403402	SUBOXONE	Tablet
63629403403	SUBOXONE	Tablet
63629409201	SUBUTEX	Tablet
63629409202	SUBUTEX	Tablet
63629507401	BUPRENORPHINE-NALOXONE	Tablet
63629712501	BUPRENORPHINE HYDROCHLORIDE	Tablet
63629712502	BUPRENORPHINE HYDROCHLORIDE	Tablet
63629712503	BUPRENORPHINE HYDROCHLORIDE	Tablet
63629712504	BUPRENORPHINE HYDROCHLORIDE	Tablet
63629712505	BUPRENORPHINE HYDROCHLORIDE	Tablet
63629712506	BUPRENORPHINE HYDROCHLORIDE	Tablet
63629712507	BUPRENORPHINE HYDROCHLORIDE	Tablet
63629712601	BUPRENORPHINE HYDROCHLORIDE	Tablet
63629712602	BUPRENORPHINE HYDROCHLORIDE	Tablet
63629712603	BUPRENORPHINE HYDROCHLORIDE	Tablet
63629712604	BUPRENORPHINE HYDROCHLORIDE	Tablet
63629712605	BUPRENORPHINE HYDROCHLORIDE	Tablet
63629712606	BUPRENORPHINE HYDROCHLORIDE	Tablet
63629712607	BUPRENORPHINE HYDROCHLORIDE	Tablet
63629712608	BUPRENORPHINE HYDROCHLORIDE	Tablet
63629727001	BUPRENORPHINE-NALOXONE	Tablet
63629727002	BUPRENORPHINE-NALOXONE	Tablet
63874108403	SUBOXONE	Tablet

© 2023 Taylor EA al. *JAMA Network Open.*

NDC	Drug Name	Formulation
63874108503	SUBOXONE	Tablet
63874117303	SUBUTEX	Tablet
63874117403	SUBUTEX	Tablet
64725093003	BUPRENORPHINE HYDROCHLORIDE	Tablet
64725093004	BUPRENORPHINE HYDROCHLORIDE	Tablet
64725192403	BUPRENORPHINE HYDROCHLORIDE	Tablet
64725192404	BUPRENORPHINE HYDROCHLORIDE	Tablet
65162041503	BUPRENORPHINE-NALOXONE	Tablet
65162041509	BUPRENORPHINE-NALOXONE	Tablet
65162041603	BUPRENORPHINE-NALOXONE	Tablet
65162041609	BUPRENORPHINE-NALOXONE	Tablet
66336001530	SUBOXONE	Tablet
66336001630	SUBOXONE	Tablet
68071138003	SUBOXONE	Tablet
68071151003	SUBOXONE	Tablet
68258299103	BUPRENORPHINE HYDROCHLORIDE	Tablet
68258299903	SUBOXONE	Tablet
68308020230	BUPRENORPHINE HYDROCHLORIDE	Tablet
68308020830	BUPRENORPHINE HYDROCHLORIDE	Tablet
70518155700	BUPRENORPHINE	Tablet
71335035301	BUPRENORPHINE HYDROCHLORIDE	Tablet
71335035302	BUPRENORPHINE HYDROCHLORIDE	Tablet
71335035303	BUPRENORPHINE HYDROCHLORIDE	Tablet
71335035304	BUPRENORPHINE HYDROCHLORIDE	Tablet
71335035305	BUPRENORPHINE HYDROCHLORIDE	Tablet
71335035306	BUPRENORPHINE HYDROCHLORIDE	Tablet
71335035307	BUPRENORPHINE HYDROCHLORIDE	Tablet
71335115403	BUPRENORPHINE	Tablet
76519117000	BUPRENORPHINE HYDROCHLORIDE	Tablet
76519117001	BUPRENORPHINE HYDROCHLORIDE	Tablet
76519117002	BUPRENORPHINE HYDROCHLORIDE	Tablet
76519117003	BUPRENORPHINE HYDROCHLORIDE	Tablet
76519117004	BUPRENORPHINE HYDROCHLORIDE	Tablet
76519117005	BUPRENORPHINE HYDROCHLORIDE	Tablet

© 2023 Taylor EA al. *JAMA Network Open.*

eTable 2. Methadone Dispensing Rates per 1000 MA Enrollees, by Dispensing Type

	National	Within OTP	Take-Home
Q1 2020	0.976	0.837	0.139
Q2 2020	1.163	0.967	0.196
Q3 2020	1.961	1.712	0.25
Q4 2020	2.579	2.252	0.327
Q1 2021	3.629	3.176	0.453
Q2 2021	4.261	3.755	0.506
Q3 2021	4.519	4.014	0.505
Q4 2021	4.878	4.342	0.536
Q1 2022	4.711	4.17	0.541

© 2023 Taylor EA al. *JAMA Network Open.*

eTable 3. Methadone Dispensing Rates per 1000 MA Enrollees, by Age and Dual Eligibility Status

	65 and Over		Under 65	
	**Dual**	**Other/Unknown**	**Dual**	**Other/Unknown**
Q1 2020	2.698	0.247	9.387	1.978
Q2 2020	3.344	0.247	11.911	2.665
Q3 2020	5.726	0.383	18.492	5.155
Q4 2020	6.961	0.479	25.159	7.683
Q1 2021	10.17	0.742	34.186	11.493
Q2 2021	11.8	0.84	39.525	12.917
Q3 2021	12.495	0.879	43.475	13.127
Q4 2021	13.894	0.958	47.018	13.982
Q1 2022	10.509	1.009	43.83	13.452

© 2023 Taylor EA al. *JAMA Network Open.*

eTable 4. Rates of Buprenorphine Dispensed From Pharmacies per 1000 MA Enrollees, by Age

	**National**	**Beneficiaries 65+**	**Beneficiaries <65**
2019 Q1	4.64	1.526	31.679
2019 Q2	5.093	1.64	34.48
2019 Q3	5.378	1.733	35.88
2019 Q4	5.706	1.809	38.128
2020 Q1	6.204	2.189	40.848
2020 Q2	6.38	2.204	41.902
2020 Q3	6.752	2.336	43.682
2020 Q4	6.947	2.417	44.621
2021 Q1	6.89	2.608	44.909
2021 Q2	7.301	2.726	47.231
2021 Q3	7.707	2.87	49.395
2021 Q4	7.789	2.894	50.084
2022 Q1	7.448	2.923	50.001

© 2023 Taylor EA al. *JAMA Network Open.*
