# Supplementary material for: Trends in Methadone Dispensing for Opioid Use Disorder After Medicare Payment Policy Changes
Source: JAMA Netw Open. 2023 May 19;6(5):e2314328. doi: 10.1001/jamanetworkopen.2023.14328 (PMC10199341; doi:10.1001/jamanetworkopen.2023.14328)
Supplement: Supplement 2. — Data Sharing Statement [file jamanetwopen-e2314328-s002.pdf]

Data Sharing Statement

Taylor. Trends in Methadone Dispensing for Opioid Use Disorder After Medicare Payment Policy Changes. *JAMA Netw Open*. Published May 19, 2023.
doi:10.1001/jamanetworkopen.2023.14328

Data
Data available: No

Additional Information
Explanation for why data not available: Our data were accessed as part of a data use agreement with the data owner, which prohibits access to the data for those not on the original agreement.
